# Stability Analysis and Structure Optimization of Unequal-Pitch End Mills

**DOI:** 10.3390/ma14227003

**Published:** 2021-11-19

**Authors:** Wanying Nie, Minli Zheng, Shicheng Xu, Yuexiu Liu, Haibin Yu

**Affiliations:** 1 College of Mechanical and Power Engineering, Harbin University of Science and Technology, Harbin 150080, China; jane9312@163.com (W.N.); 1920110080@stu.hrbust.edu.cn (S.X.); Yuexiu6@outlook.com (Y.L.); Quentina_YU@hrbust.edu.cn (H.Y.); 2 Key Laboratory of Advanced Manufacturing and Intelligent Technology, Ministry of Education, Harbin 150080, China

**Keywords:** unequal-pitch end mill, cutting stability, dynamic balance, groove shape, dynamic performance

## Abstract

The damping performance of unequal tooth milling cutters is controlled by the pitch parameters. How to improve the vibration damping and dynamic balance of milling cutters needs to be further studied. This paper analyzes the pitch angle through the stability of the lobe diagram and the spectral characteristics, and unequal-pitch end mills with asymmetric structure were determined to have better cutting stability. Due to the principle error of the asymmetrical tool, dynamic balance accuracy is poor. The dynamic balance of the tool is analyzed, and the centroid model of the tool is established. In order to improve the dynamic balance accuracy of tools, the parameters of the groove shape are analyzed and optimized, and balance accuracy is improved. Through modal and milling-force analysis, the relative vibration displacement and cutting force of the optimized tool were reduced by 17% and 10%, respectively, which determined that such tools have better dynamic performance. Here, unequal tooth end mills could reduce vibration and had higher accuracy in dynamic balance by adjusting the parameters of the pitch angles and chip pockets, so that the tool could have higher cutting stability.

## 1. Introduction

With the continuous advancement of the cutting technology, machining and manufacturing are gradually developing towards high efficiency, speed, and quality. Processing difficulty for key components increases, and the consistency of the surface quality of the workpiece is always affected by its vibration. In order to suppress frequent vibrations during the cutting process, Slavicek [1] first proposed the design concept of the uneven distribution of the pitch angle in the 1960s. Shirase et al. [2] observed that unequal-pitch cutters have great vibration reduction properties by compared the cutting performance of ordinary and unequal-pitch cutters. Budak and Ahmad et al. [3,4] developed dynamic models of unequal-pitch end mills, explored the cutting stability of unequal-pitch cutters, and proposed a method to optimize pitch angles. N. Olgac et al. [5] explained that an unequal-pitch cutter can suppress cutting chatter from both the physical and the mathematical perspective. Sellmeier et al. [6] presented a model of cutting force and surface machining error for unequal-pitch end mills, and observed that unequal-pitch end mills can both suppress chatter and effectively reduce machining surface error. Huang and Jin [7,8] developed dynamic models of unequal-pitch cutters by analyzing machining stability, and optimized the structure of the cutters and cutting parameters in the milling process. Zhou et al. [9] developed a theoretical model of cutting forces for unequal-pitch end mills, and observed the reliability of the model through simulations and experiments. Chen [10] analyzed the influence of tool geometry parameters on the cutting process by the 3D modeling of unequal-pitch end mills. Otto et al. [11] presented the cutting stability of nonuniform-pitch and variable-helix tools, and cutting parameters were optimized by this method. Guo et al. [12] developed the stability lobe diagram for analyzing the chatter modeling of nonuniform helix tools, and the damping property of these tools was fully proven.

The dynamic balance of the tool determines the centrifugal force and vibration degree in the cutting process, which directly affects cutting performance and tool life, and it is a key issue in the design and manufacture of cutters. The requirements of dynamic balance for rotating tools are even stricter in high-speed cutting. Liu and Xie et al. [13,14] developed the tool centrifugal force to determine the balance quality level in high-speed finishing. Zhao [15] developed the relationship between residual rotor unbalance and tool dynamic balance, and analyzed the development basis of the balance level. Wang et al. [16] developed the critical instability speed of the tool by analyzing the dynamic balance. Zhang et al. [17] verified the influence of the tool dynamic balance on the surface roughness and machining accuracy of the workpiece through cutting experiments. Tang et al. [18] analyzed the influence of geometric parameters for three-edge end mills of unequal pitch on dynamic balance. The identification and prediction of the parameters affect the accuracy analysis of the dynamic balance, which is greatly significant to the cutting performance of the system. Yan et al. [19] regarded dynamic unbalance as a modal parameter of the shaft system, and used modal analysis technology to identify the dynamic balance parameters of the rotor. Qin et al. [20] proposed a new virtual vibration system on the instantaneous center method for dynamic balance measurement, and combined it with experiments to show that the system could measure the dynamic balance with high precision. The unbalance correction of the rotor now mainly adopts the method of removal weight or counter weight to compensate for its unbalance accuracy. Chen [21] adopted a real-time counterweight of the holder to achieve tool balance, and determined the quality level of ultrahigh precision balance. Lu [22] developed the balance model of the HSK tool holder with UG software. Zeng and Zhang [23,24] simplified the model for forming milling deduplication in a V shape and circumference.

There is a theoretical basis for the damping mechanism of unequal-pitch milling cutters and the dynamic balance of rotating tools, but there is still a lack of research about the dynamic balance of unequal-pitch end mills. This paper combines stability and dynamic balance analysis of unequal tooth end mills to determine the structural parameters of the end mill, and the centroid model of the tool is established. An unequal tooth end mill is optimized on the basis of the characteristics of the groove shape. At the same time, the tool modal and milling forces are analyzed, and the dynamic performance of the optimized tool is determined to improve cutting stability while meeting tool design criteria. In this paper, the vibration reduction effect of asymmetric milling cutters is evaluated, and the chip flute parameters of milling cutters are optimized by improving the accuracy of dynamic balance. This can theoretically alleviate chatter, meet the dynamic balance accuracy in actual processing, the centrifugal force is reduced, and cutting stability is further improved.

## 2. Analysis of Structural Parameters of Unequal-Pitch End Mills

### 2.1. Stability Analysis of Unequal-Pitch End Mills

The pitch angle distribution of an end mill is a key factor affecting the system time delay, which ultimately determines stable cutting limit conditions. The milling force model of the end mill is as follows.

(1) $$\begin{array}{l} F_{tj}(t)=K_{t}h\left(\phi_{j}\left(t\right)\right)a_{p}\\ F_{rj}(t)=K_{r}h\left(\phi_{j}\left(t\right)\right)a_{p} \end{array}$$

where *K_t_* and *K_r_* are the tangential and radial milling forces coefficients, respectively; $\phi_{j}\left(t\right)$ is the contact angle of tooth *j*; $h\left(\phi_{j}\left(t\right)\right)$ is the cutting thickness; *a_p_* is the cutting depth; and $F_{tj}\left(t\right)$ and $F_{tj}\left(t\right)$ are the tangential and radial milling forces, respectively.

According to the mechanism of regenerative chatter, the chatter of the system is mainly caused by the cutting dynamic excitation force, which is directly related to the dynamic cutting thickness of the milling cutter. The equation is as follows.

(2) $$\Delta h_{j}\left(t\right)=\left[\Delta x_{j}\sin\phi_{j}\left(t\right)+\Delta y_{j}\cos\phi_{j}\left(t\right)\right]$$

where Δx and Δy are vibration displacements. Therefore, the milling force model of the variable-pitch end mill is

(3) $$F\left(t\right)=\sum_{j=1}^{N}\frac{1}{2}a_{p}K_{t}\left[A_{j}\left(t\right)\right]\left\{\Delta_{j}\left(t\right)\right\}$$

The dynamic model of variable-pitch end mill is

(4) $$\left\{F\left(t\right)\right\}e^{iw_{c}t}=\frac{1}{2}K_{t}a_{p}\sum_{j=1}^{N}\left[A_{0}\right]\left(1-e^{-iw_{c}\tau_{j}}\right)\left[G\left(iw_{c}\right)\right]\left\{F\left(t\right)\right\}e^{iw_{c}t}$$

where $\left[A_{0}\right]$ is the time-varying coefficient matrix, and $G\left(iw_{c}\right)$ is the frequency domain transfer function.

The limit cutting depth is

(5) $$a_{p\lim}^{vp}=-\frac{4\pi }{K_{t}}\frac{\lambda_{I}}{\sum_{j=1}^{N}\sin\left(w_{c}T_{j}\right)}=-\frac{4\pi }{K_{t}}\frac{\lambda_{I}}{S}$$

**Figure 1:**
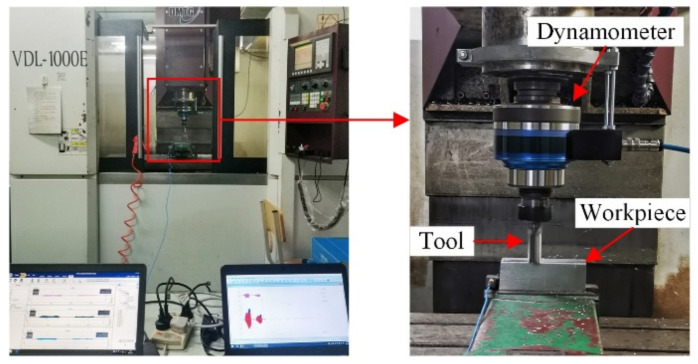
Field test.

**Figure 2:**
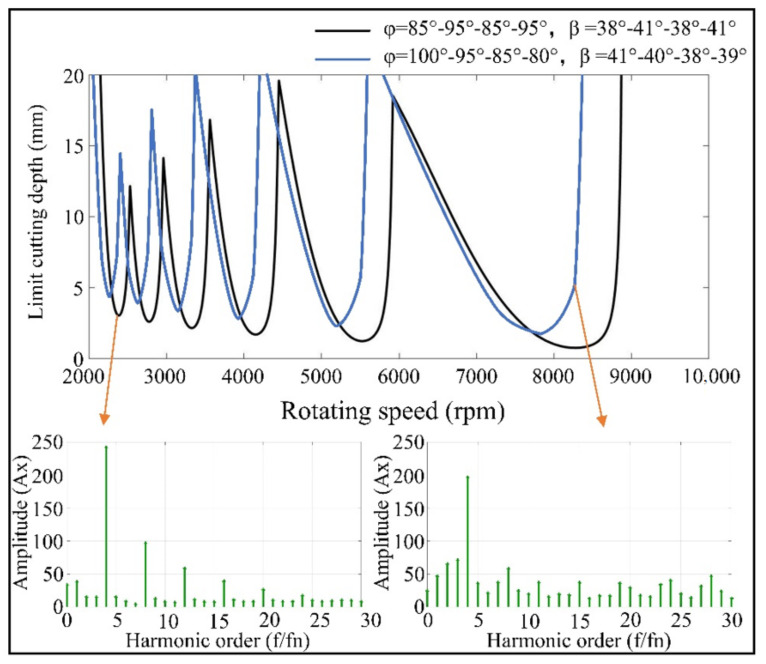
Lobes and spectrum in tool comparison.

Combined with the aluminum alloy milling test (as shown in Figure 1), the testing plan is shown in Table 1. The basic parameters of the processing system were determined: natural frequency *w_nx_* = 1047 Hz, *w_nx_* = 855 Hz, stiffness *k_x_* = 2.825 × 10^7^, *k_y_* = 9.319 × 10^6^ and damping coefficient *ζ_x_* = 0.0323, *ζ_y_* = 0.0604. The milling force coefficients of aluminum alloy materials are *K_t_* = 860 Mpa and *K_r_* = 355 Mpa. In cutting stability, the lobe diagram was drawn, and the milling excitation force was spectrally analyzed. Tool stability was analyzed from the perspectives of milling chatter and forced vibration. Figure 2 shows the lobes and spectrum, where the tool of the asymmetrical pitch had a larger limit cutting depth, and its frequency spectrum was flatter, indicating that this type of tool has better vibration damping performance.

### 2.2. Dynamic Balance Analysis of Unequal-Pitch End Mills

Under the condition of the same milling parameters, due to the uneven material organization and the asymmetrical structure, the main inertial axis passing through the cutter centroid did not coincide with the axis of the rotation, resulting in eccentricity and unbalance (Equation (6)). The centrifugal force was affected by the speed and eccentricity in Equation (7). The cutter had a small eccentricity, and excessive centrifugal force is generated in the high-speed milling. The increase in eccentricity causes the tool to withstand periodic excitation forces, which directly affects the vibration and tool wear, and reduces tool life and cutting efficiency. Therefore, cutter eccentricity should be minimized to ensure the accuracy of the dynamic balance in the design stage.

(6) U=me

(7) $$F=mew^{2}=me{\left(\frac{\pi n}{30}\right)}^{2}$$

where *U* is the rotor unbalance (g·mm); *m* is the rotor mass (kg); *e* is the deviation of the rotor’s gravity center from the rotation axis, that is, the eccentricity (um); *F* is the centrifugal force (N); *w* is the angular velocity of the rotor (rad/s); and *n* is the rotational speed of the rotor (r/min).

The larger the cutter mass is, the larger the allowable residual unbalance usually is. ISO1940-1 Quality Requirements for Dynamic Balance of Rigid Rotors specifies that the quality of balance is a dynamic concept that is represented by a set of discrete 45° diagonal lines on the standard level diagram of the balance quality; the relation is as shown in Equation (8). In order to determine the balance quality level and solve dynamic balance problems, the eccentricity needs to be defined regarding its size, orientation, and axial distribution. Therefore, it is necessary to analyze the centroid of unequal-pitch end mills.

(8) $$G=e_{per}\times w$$

where *G* is the balance quality level, and *e_per_* is the allowable eccentricity (the allowable residual unbalance per unit mass).

**Figure 3:**
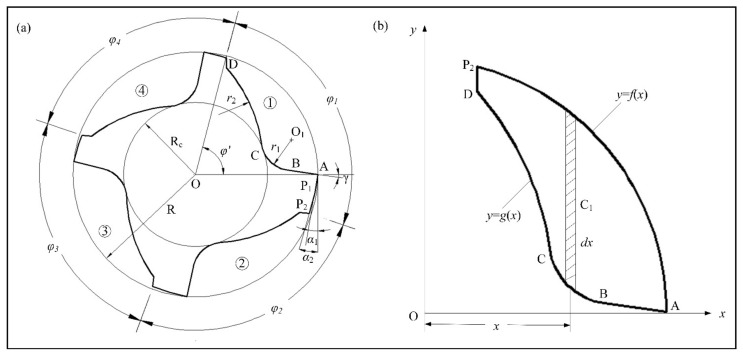
End section of variable-pitch end mill: (**a**) parameter characteristics with four flutes; (**b**) schematic of calculating centroid coordinates.

The structural design of an unequal-pitch end mill is mainly for the determination of tool geometric parameters (as shown in Figure 3a). The cutter radius is *R*; the radius of the core thickness is *R_c_*; and pitch angles are *φ*_1_, *φ*_2_, *φ*_3_, *φ*_4_. The four chip pockets are processed with the same grinding wheel and grinding parameters, and except for manufacturing defects, the shapes are almost the same. The related dimensional parameters of the peripheral tooth are mainly the chip pocket and the flank. The geometry of the chip pocket includes rake surface curve AB, groove bottom arc BC, and tooth back arc CD. The flank surface structures are mainly first flank AP_1_, second flank P_1_P_2_, and tooth back boss DP_2_.

Combined with the end sectional profile of the unequal-pitch end mill, the structure of the most removed material was the chip pocket, and its effect was the most obvious on the eccentricity of the end mill. The parameters of the section shape of the end mill were determined, and the mathematical model of the end section was developed by designing its geometric structure. The structural feature of the chip pocket was composed of a straight line and two arcs. The rake angle is γ, the arc of the groove bottom was tangential to the rake face at point B, and the two arcs of the chip pocket were tangential to the core circle at point C. The center of the arc of the groove bottom is O_1_ (*x*_1_, *y*_1_), the radius is *r*_1_, the center of the tooth back arc is O_2_ (*x*_2_, *y*_2_) and the radius is *r*_2_. Then, the mathematical model of the chip pocket is

(9) $$\left\{\begin{array}{l} y=\left(x-R\right)\tan\gamma \\ {\left(x-x_{io}\right)}^{2}+{\left(y-y_{io}\right)}^{2}=r_{i}{}^{2}\;\;i=1.2 \end{array}\right.$$

where *x_io_*, *y_io,_* and *r_i_* are the abscissa, ordinate, and radius of the arc center, respectively.

### 2.3. Centroid Equation of Unequal-Pitch End Mills

This paper ignores the structure of the flanks and adopts the method shown in Figure 3b to define the coordinate system of the end section. The inner and outer contour equations of the section curve are defined as $y=g\left(x\right)$ and $y=f\left(x\right)$; the former is the mathematical model of the end section of the cutter, and the latter is the equation of the outer diameter circle of the cutter. The microelement of width *dx* in parallel to the *y* axis was selected on the section. When the density of the end mill is uniform, the coordinate of centroid position C_i_ of the microelement is (*x_i_*, $\frac{f\left(x_{i}\right)+g\left(x_{i}\right)}{2}$).

Since the shape and size of the chip pocket of each cutter tooth are equal, the coordinates of the centroid in the *i*+1th tooth are

(10) $$\left[\begin{array}{l} x_{i+1}\\ y_{i+1} \end{array}\right]=\left[\begin{array}{l} \cos\varphi_{i+1}\;-\sin\varphi_{i+1}\\ \sin\varphi_{i+1}\;\cos\varphi_{i+1} \end{array}\right]\left[\begin{array}{l} x_{i}\\ y_{i} \end{array}\right]\;i=1\;,\;2\;....$$

where: $\varphi_{i}$ is the angle between teeth *i* and *i* + 1, so $\sum_{i=1}^{n}\varphi_{i}=2\pi$.

When the number of the teeth of the unequal pitch end mill is *n*, the centroid coordinates (*x*_0_, *y*_0_) of the end section [18] are

(11) $$\begin{array}{l} x_{0}=-\frac{\sum_{i=1}^{n}x_{i}S_{i}}{\pi R^{2}-\sum_{i=1}^{n}S_{i}}\\ y_{0}=-\frac{\sum_{i=1}^{n}y_{i}S_{i}}{\pi R^{2}-\sum_{i=1}^{n}S_{i}} \end{array}$$

(12) $$e_{0}=\sqrt{x_{0}{}^{2}+y_{0}{}^{2}}$$

where *S_i_*, *x_i,_* and *y_i_* are the end face area and the centroid coordinate of the *i* tooth, respectively.

**Figure 4:**
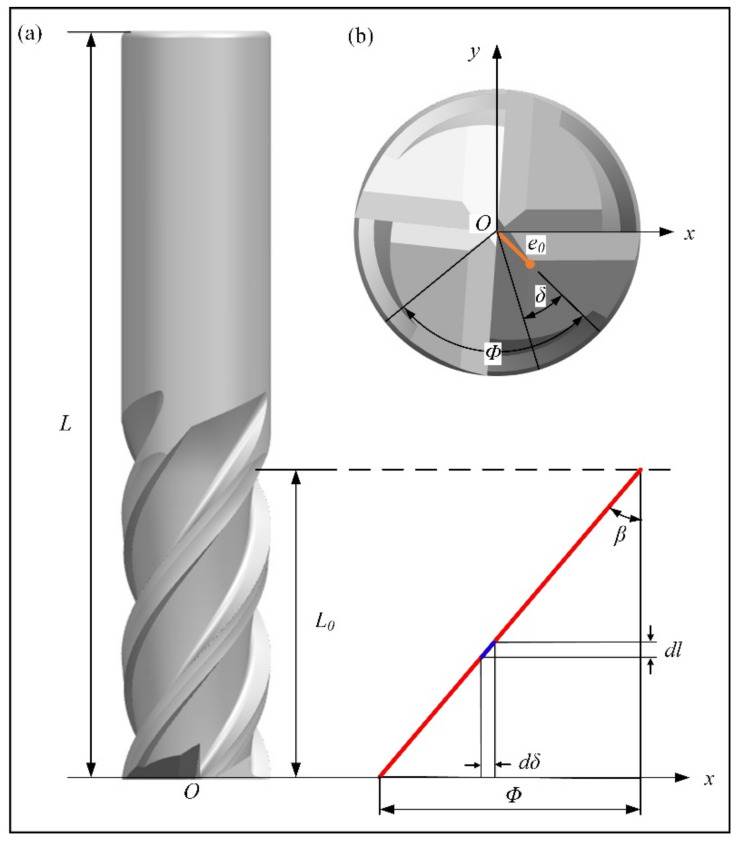
Relationship diagram of eccentricity for unequal-pitch end mill: (**a**) axial; (**b**) radial.

When the edge length of the end mill is *L*_0_, total length is *L*, and the helix angle is *β*. Microelement *dl* was selected at the effective edge length of the end mill, the equivalent mass was dm=ρVdl, and the phase angle of the centroid was *δ* (as in Figure 4). The chip pocket of the end mill spirally rose, and the eccentricity of the end section *e*_0_ rotated by phase angle Φ along the spiral edge line, so the phase angle was $\Phi =\frac{l\tan\beta }{R}$ at edge length *l*.

When end mills have unequal pitch and equal helix angle, the effective coordinate of the centroid at edge length *l* is

(13) $$\left[\begin{array}{c} x_{l}\\ y_{l} \end{array}\right]=\frac{\int_{0}^{\Phi }\rho Vdle_{0}}{\rho Vl}\left[\begin{array}{c} \cos\delta \\ \sin\delta \end{array}\right]=\frac{Re_{0}}{l\tan\beta }\left[\begin{array}{c} \sin\Phi \\ 1-\cos\Phi \end{array}\right]=\frac{e_{0}}{\Phi }\left[\begin{array}{c} \sin\Phi \\ 1-\cos\Phi \end{array}\right]$$

**Figure 5:**
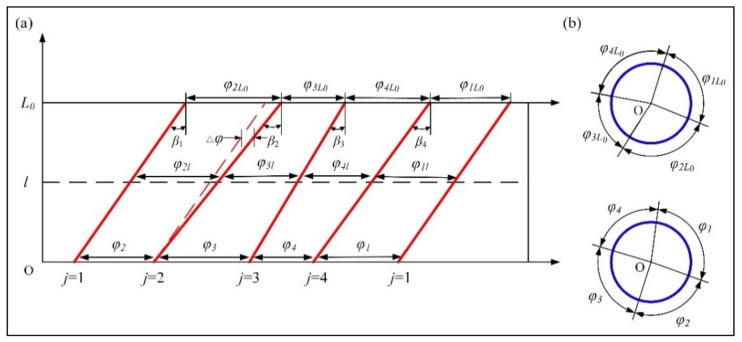
Expanded diagram of the cutting edge for unequal-pitch end mill: (**a**) axial expansion of cutting edge; (**b**) radial distribution of pitch angle.

When the helix angle of each edge line is different, pitch angles vary linearly (cylindrical end mills) or exponentially (conical end mills) from the front to the end of the cutting edge. Figure 5a shows the axial expansion of the cutting edge for the cylindrical end mill, and the pitch angles of any section are different in the axial direction. Figure 5b shows the radical distribution of the pitch angle at the beginning and the end of the cutting edge. Therefore, when the eccentricity of each end section for the unequal-pitch end mill is defined, it is necessary to combine helix angle *β* and length *l* to define the centroid position (*x_il_*, *y_il_*) of each edge line in the section.

According to the axial expansion diagram of the unequal-pitch end mill, with a change in edge length *l*, pitch angle difference ∆*φ* of the end section is

(14) $$\Delta \varphi_{i}=\frac{2l}{D}\left(\tan\beta_{i}-\tan\beta_{i-1}\right)$$

At edge length *l*, the pitch angle in edge *i* is

(15) $$\varphi_{il}=\varphi_{i0}+\frac{2l}{D}\left(\tan\beta_{i}-\tan\beta_{i-1}\right)$$

Therefore, the centroid (*x_ol_*, *y_ol_*) at edge length *l* is

(16) $$\left[\begin{array}{l} x_{ol}\\ y_{ol} \end{array}\right]=\frac{-S}{\pi R^{2}-nS}\left(1+\sum_{i=2}^{n}T_{i}\right)\left[\begin{array}{l} x_{1l}\\ y_{1l} \end{array}\right]=\frac{-SR}{l\tan\beta_{1}\left(\pi R^{2}-nS\right)}\left(1+\sum_{i=2}^{n}T_{i}\right)\left[\begin{array}{c} \sin\Phi \\ 1-\cos\Phi \end{array}\right]\left[\begin{array}{c} x_{1}\\ y_{1} \end{array}\right]$$

among them $T_{i}=\left[\begin{array}{cc} \cos\sum_{j=2}^{j=i}\varphi_{j} & -\sin\sum_{j=2}^{j=i}\varphi_{j}\\ \sin\sum_{j=2}^{j=i}\varphi_{j} & \cos\sum_{j=2}^{j=i}\varphi_{j} \end{array}\right]$.

By analyzing the dynamic balance of unequal-pitch end mills, the mathematical model of the end mill and the equation of the centroid are defined, and the relationship between the geometric parameters of the tool and the accuracy of the dynamic balance is developed. The asymmetric geometric characteristics of unequal-pitch end mills seriously affect eccentricity, which becomes a key factor that determines the stable cutting of the tool in a high-speed milling process. Therefore, it is necessary to design and optimize the structure of unequal-pitch end mills on the basis of dynamic balance problems.

## 3. Profile Optimization of Unequal-Pitch End Mills

### 3.1. Optimization Method

The unbalance correction of the rotor generally needs to be combined with the dynamic balance test to identify the unbalance parameters. The sum of the inertial centrifugal forces (unbalance vector) close to zero is through the method of weight removal or counterweight compensation. There are multiple trial attempts in this process, and the compensation time of the correction is longer. When unequal-pitch end mills have principle eccentricity error in the design stage, the optimized structure of the end mill can reduce the tool eccentricity to meet the balance quality level, decrease the number of the multiple compensation instances in the dynamic balance test, and improve correction accuracy and balance efficiency.

**Figure 6:**
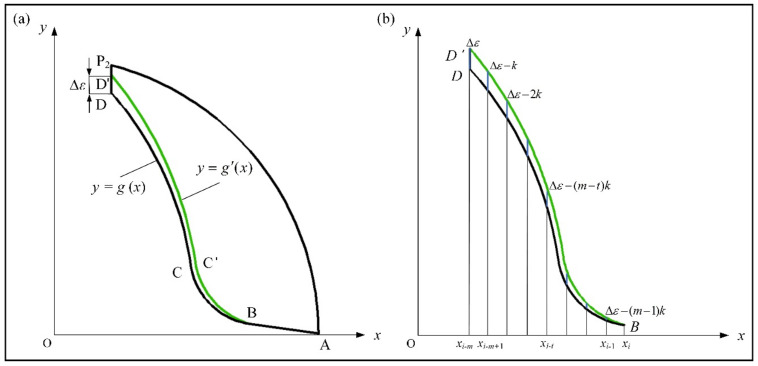
Optimization for variable-pitch end mill: (**a**) groove morphology; (**b**) comparison before and after optimization.

On the basis of the centroid equation of the unequal-pitch end mill, the mathematical model of the section is analyzed, the geometry of the end mill is optimized, and the eccentricity of the tool is reduced to meet evaluation standard *e*_0_ < [*e*]. The chip pocket is the key factor affecting dynamic balance, and its design parameters are used as the characteristics for the optimization. The optimized method of changing the groove parameters enables end mills to reach the theoretical balance accuracy. This paper adopted the unbalance adjustment method of component correction, and the *x* and *y* axes of the coordinate system were fixed, so that optimization analysis is simpler, and the error is small. The optimization method is shown in Figure 6a, where rake face *AB* remained unchanged, and the parameters of chip pocket BD were adjusted, that is, the mathematical model of the groove shape *g*(*x*) became *g’*(*x*). This method better reduces the eccentricity of the tool without affecting the parameters of the tool rake angle and pitch angles.

The comparison diagram before and after optimization of the groove shape is shown in Figure 6b. The discrete analysis method was adopted to divide the groove shape into length Δx in interval [*x_B_*, *x_D_*], *l_BD_* = *x_B_* − *x_D_* = *m*, where *m* is an integer as the estimated parameter for the horizontal distance of the groove shape. The mathematic model of the groove shape is $g\left(x_{t}\right)=y_{t}$, the model after optimization is $g^{\prime }\left(x_{t}\right)=y_{t}+\Delta \varepsilon -\left(m-t\right)k$, where $k=\frac{\Delta \varepsilon }{m}$, Δε is the maximal displacement of the adjustment for the chip pocket; by increasing in *x*, the movement displacement of the groove gradually becomes smaller, and the groove lastly coincides with tangent point B.

### 3.2. Optimization of Unequal-Pitch End Mills

According to the optimization method of the unequal-pitch end mill, the spiral groove depth is adjusted, and the sectional profile is optimized to reach a reduction in eccentricity. When the pitch angle of the unequal-pitch end mill is (90 − 2θ)° − (90 − θ)° − (90 + θ)° − (90 + 2θ)°, the design parameters of the chip pocket are consistent and satisfy the design requirements of the tool strength. Pitch angles are substituted into eccentricity calculation Equation (12) to obtain eccentricity *e*_0_ of the end mill in the end section, as in Equation (17), which shows that, when the pitch angle difference is 0, that is, the equal pitch, there is theoretically no eccentricity in the end section of the tool, and with increasing the pitch angle difference, the eccentricity is greater.

(17) $$\begin{array}{ll} e_{0} & =\frac{S_{1}}{\pi R^{2}-4S_{1}}\sqrt{2\left(x_{1}{}^{2}+y_{1}{}^{2}\right)\left(1-\cos3\theta \right)}\\ & =\frac{1}{\pi R^{2}-4S_{1}}\sqrt{\left[2{\left(\int \left(f\left(x\right)-g\left(x\right)\right)xdx\right)}^{2}-\frac{1}{2}{\left(\int \left(f^{2}\left(x\right)-g^{2}\left(x\right)\right)dx\right)}^{2}\right]\left(1-\cos3\theta \right)} \end{array}$$

When the diameter of the unequal pitch end mill is 20 mm, the core thickness is 12 mm, the pitch angle is 100°–95°–85°–80°, and the helix angle is 41°–40°–38°–39°. On the basis of the centroid equation of the unequal-pitch end mill, the geometric structure parameters of the end mill are substituted into Equations (15)–(17). Results show that, with increasing the axial edge length, pitch angle difference *θ* of the end section becomes smaller, and the eccentricity of the tool gradually decreases. Therefore, optimization mainly analyzes the end section of the end mill at *l* = 0, and the centroid at this position is calculated as (*x*_0_, *y*_0_) = (0.077 mm, −0.129 mm), that is, *e*_0_ = 0.150 mm = 150 um.

In the process of tool design, if the balance quality level is too low, the machine tool spindle is adversely affected, but if it is too high, the cutting is subject to technical and economic constraints. Therefore, the balance quality level should be reasonably selected. In this paper, the design criteria for the eccentricity of unequal-pitch end mills were determined by the dynamic balance experiment of a conventional end mill, and the result was used as the minimal eccentricity [*e*] of the end mill. The experimental tool is a symmetrical unequal-pitch end mill with 85°–95°–85°–95°pitch angle and 38°–41°–38°–41° helix angle; other parameters were consistent with the optimized tool structure in this paper. The experiment was set at *n* = 5000 r/min, and the result was *e_max_* = 10 um. When the design criterion of the eccentricity was [*e*] = 10 um, the balance quality level of the high-speed cutting tool was G2.5.

**Figure 7:**
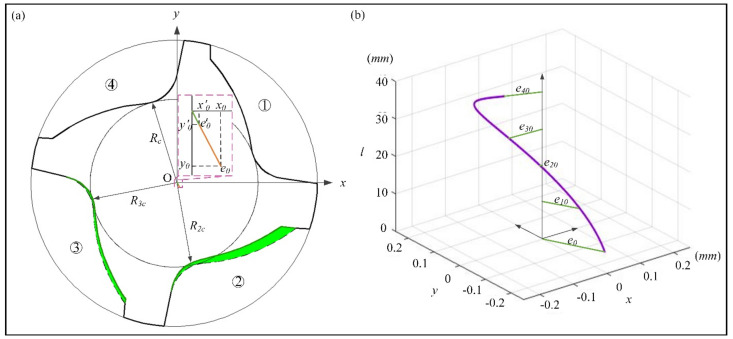
Centroid distribution of optimized variable pitch end mill: (**a**) end section; (**b**) cutting edge axial.

The design benchmark of the eccentricity was determined through experiments. According to the design parameters of the above-mentioned unequal-pitch end mill, maximal eccentricity was 150 um > [*e*] = 10 um, and the corresponding level of the balance quality was G40, which coilf noy meet the dynamic balance accuracy of the high-speed milling tool. The section shape of the end mill was optimized, combined with the optimization method of this paper. Figure 7 shows the optimized profile of the end section, with tool core thickness *R_c_* = 6 mm, optimized sectional groove shape with *R_2c_* = 5.7 mm, *R_3c_* = 5.9 mm, $\Delta \varepsilon_{2}=1.2\mathrm{mm}$ and $\Delta \varepsilon_{3}=0.3\mathrm{mm}$. The final centroid was (*x*_0_′, *y*_0_′) = (0.0029, 0.0039), *e*_0_ = 0.003 mm = 3 um. When the speed was as high as 10,000 r/min, the balance accuracy of the tool could still reach G2.5, and this shows that the optimized tool meets the design requirements for dynamic balance accuracy.

## 4. Analysis of Dynamic Performance

The dynamic performance of the machining system determines the degree of vibration; the dynamic balance of the tool is closely related to the dynamic performance of the system, resulting in the cutter unbalance directly affecting the evolution trend of the milling vibration. In this paper, the structure of the tool was optimized on the basis of the minimal eccentricity to achieve the requirements of the dynamic balance accuracy. Dynamic analysis of different end mills was carried out, combined with the finite-element dynamic model. The influence of pitch angles and groove parameters was analyzed on the mode parameters and the cutting forces. There were certain differences regarding the modal between machining system and tool. However, in an actual machining process, only the tool needs to be replaced, and other parameters of the system are constant. The end mill is the section with less rigidity and the weakest vibration resistance in the whole machining system. Therefore, vibration characteristics of the end mill can be studied by analyzing the modal problem, which indirectly reflects the dynamic performance of the system.

**Figure 8:**
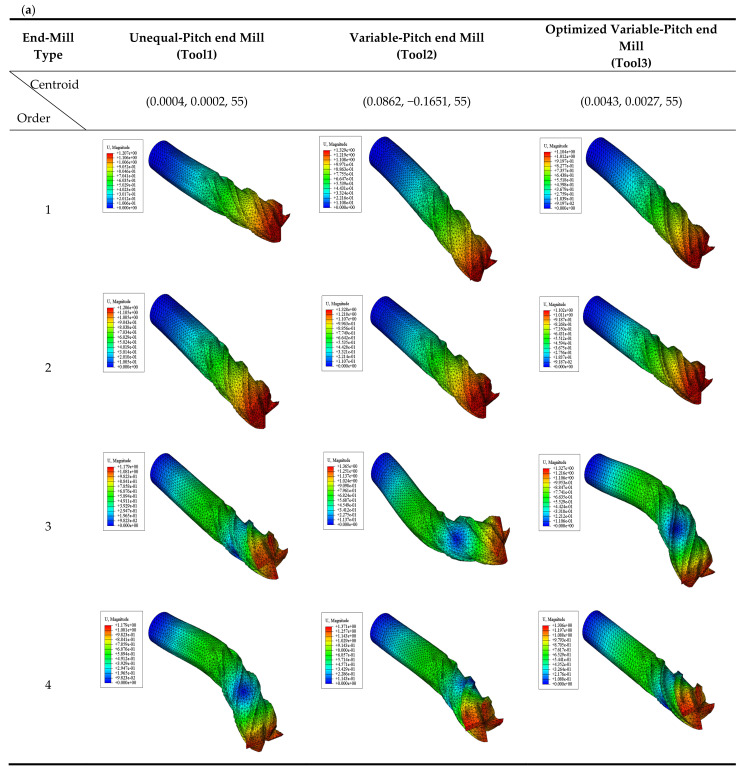

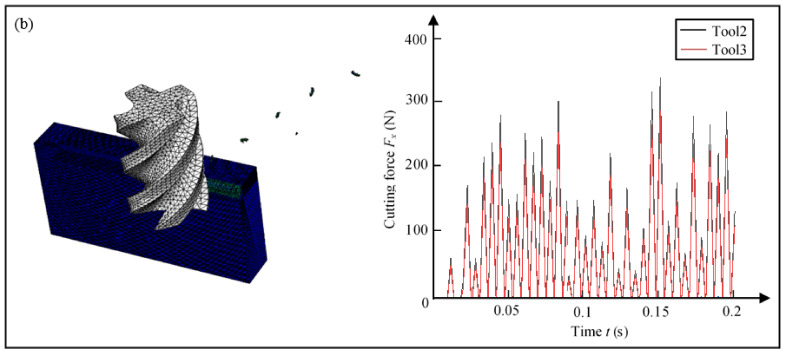
Dynamic performance for different end mills: (**a**) modal analysis; (**b**) cutting force.

In this paper, since milling vibration mainly occurred in the low- and middle-frequency regions, the first–fourth-order modal characteristics were selected for analysis. Figure 8a is the modal analysis diagram of different end mills. Table 2 shows the main modal parameters. The parametric model of the finite element caused a certain deviation of the end-mill centroid from the theoretical analysis, but it still met the design criteria of the tool. The figure shows that the change parameters in the pitch angle caused the centroid of the end mill to shift, which changed the effective mass and stiffness distribution of the tool in each order, and affected the mode shape. Modal motions bend in the first–second orders, and torsion in the third–fourth orders. The vibration of the end mill is the result of the superposition of various mode orders in the milling process. Among them, bending and torsion are the overall reflection of the vibration displacement in the low order, and more energy is stored in this range. Table 2 shows that the natural frequencies of an unequal-pitch end mill have small changes in each order, but its relative displacements have a large increase rather than an equal-pitch end mill. Bending and torsional displacements increased by 10% and 16% in the low order, and eventually promoted the actual vibration displacement of the tool to increase. However, due to the same pitch angles and changed groove parameters, eccentricity and core thickness were reduced for the optimized unequal-pitch end mill. Its mode shapes and natural frequencies were affected to a certain extent, but the change trend was small, and it had little effect on the dynamic performance of the cutting system. However, the relative displacements of the modal in the bending and torsional motions of the tool were greatly reduced by 17% and 6%, which indirectly indicates that the actual vibration displacement of the tool was reduced. At the same time, under the same cutting parameters, the optimized tool cutting force was reduced by an average of 10% (as shown in Figure 8b). Therefore, the optimization of unequal-pitch end mills effectively reduces eccentricity, improves the balance quality of the tools, enhances the dynamic performance, and strengthens the vibration reduction performance of the tools. This has important guiding significance for improving cutting stability.

## 5. Conclusions

This paper, by analyzing the relationship between the pitch angle of end mills and cutting stability, determined that the asymmetrical distribution of the pitch angle (100°–95°–85°–80°) has higher limit depth and better spectral characteristics. The tool dynamic balance is determined by combining the mathematical model of the end mill and centroid equation $e_{0}=\frac{1}{\pi R^{2}-4S_{1}}\sqrt{\left[2{\left(\int \left(f\left(x\right)-g\left(x\right)\right)xdx\right)}^{2}-\frac{1}{2}{\left(\int \left(f^{2}\left(x\right)-g^{2}\left(x\right)\right)dx\right)}^{2}\right]\left(1-\cos3\theta \right)}$; the relationship between pitch angle and dynamic balance was analyzed. As pitch difference angle increased, the tool eccentricity became larger, and the dynamic balance accuracy decreased. Analyzing the end-mill groove optimization method determined that the tool profile was optimized on the basis of the minimal eccentricity criterion, so that *e*_0_ = 150 um was reduced to *e’*_0_ = 3 um, which achieved the tool design criterion of dynamic balance accuracy. On the basis of tool modal and milling simulations, the dynamic performance of the optimized tool was analyzed, and the relative vibration displacement and cutting force of the optimized tool were reduced by 17% and 10% respectively. Therefore, in actual milling, the dynamic balance problem of asymmetric milling cutters is solved with better vibration damping tool characteristics. The tool had better balance quality and better vibration damping characteristics, which promotes further improving the processing efficiency and surface quality of a workpiece.

## Figures and Tables

**Table 1:** Testing plan.

Test No.	Rotating Speed *n* (r/min)	Cutting Depth *a_p_* (mm)	Cutting Width *a_e_* (mm)	Feed Rate *f_r_* (mm/rev)
1	3000	2	5	0.2
2				0.4
3				0.6
4				0.8
5				1.0
6	4000	0.5	5	0.8
7		1.0		
8		1.5		
9		2.0		
10		2.5		

**Table 2:** Modal parameters for different end mills.

Modal Parameters		Natural Frequency *w_n_* (Hz)			Maximal Displacement of Mode U_max_ (mm)		
	Type	Tool1	Tool2	Tool3	Tool1	Tool2	Tool3
Order							
1		2419	2418	2426	1.207	1.329	1.104
2		2420	2421	2431	1.206	1.328	1.102
3		10,852	10,816	10,781	1.179	1.365	1.327
4		10,855	10,883	10,885	1.179	1.371	1.306

## Data Availability

The data presented in this study are available on request from the corresponding author.
